# Wild-type p53 binds to *MYC* promoter G-quadruplex

**DOI:** 10.1042/BSR20160232

**Published:** 2016-10-14

**Authors:** Marek Petr, Robert Helma, Alena Polášková, Aneta Krejčí, Zuzana Dvořáková, Iva Kejnovská, Lucie Navrátilová, Matej Adámik, Michaela Vorlíčková, Marie Brázdová

**Affiliations:** * Department of Biophysical Chemistry and Molecular Oncology, Institute of Biophysics, The Czech Academy of Sciences, Královopolská 135, 612 65 Brno, Czech Republic; † Department of CD Spectroscopy of Nucleic Acids, Institute of Biophysics, The Czech Academy of Sciences, Královopolská 135, 612 65 Brno, Czech Republic

**Keywords:** DNA–protein interaction, G-quadruplex, *MYC*, p53 protein

## Abstract

G-quadruplexes are four-stranded nucleic acid structures that are implicated in the regulation of transcription, translation and replication. Genome regions enriched in putative G-quadruplex motifs include telomeres and gene promoters. Tumour suppressor p53 plays a critical role in regulatory pathways leading to cell cycle arrest, DNA repair and apoptosis. In addition to transcriptional regulation mediated via sequence-specific DNA binding, p53 can selectively bind various non-B DNA structures. In the present study, wild-type p53 (wtp53) binding to G-quadruplex formed by *MYC* promoter nuclease hypersensitive element (NHE) III_1_ region was investigated. Wtp53 binding to *MYC* G-quadruplex is comparable to interaction with specific p53 consensus sequence (p53CON). Apart from the full-length wtp53, its isolated C-terminal region (aa 320–393) as well, is capable of high-affinity *MYC* G-quadruplex binding, suggesting its critical role in this type of interaction. Moreover, wtp53 binds to *MYC* promoter region containing putative G-quadruplex motif in two wtp53-expressing cell lines. The results suggest that wtp53 binding to G-quadruplexes can take part in transcriptional regulation of its target genes.

## Abstract (precis)

We found that the p53 tumour suppressor protein binds specifically to the G-quadruplex DNA formed by *MYC* promoter sequence. We propose that p53 binding to G-quadruplexes can participate in regulation of p53 target genes.

## INTRODUCTION

G-quadruplexes belong to a group of non-canonical DNA structures with suggested participation in cellular processes such as regulation of transcription and replication. G-quadruplexes are formed by at least two stacked G-tetrads which are planar arrangements of four guanines connected via Hoogsteen base pairing. G-quadruplexes are further stabilized by monovalent cations, predominantly K^+^ or Na^+^, positioned in the plane or between G-tetrads [1,2]. Putative G-quadruplex motifs in the human genome are frequently found in G/C-rich nuclease hypersensitive regions of gene promoters [3] and oncogenes contain a G-quadruplex motif more often than tumour suppressor genes [4]. Formation of G-quadruplexes in promoters has been proposed to serve a regulatory function where the G-quadruplex structure acts as a transcriptional modulator that can be targeted with potential anti-cancer drugs [5,6]. Both repression and induction of genes via promoter G-quadruplex motif have been observed [7–11]. In the majority of cases, the transcriptional repression is associated with G-quadruplex structure formation [9,11].

Many G-quadruplexes formed *in vitro* by oncogene promoter sequences have been identified (reviewed in [8,12]) and the *MYC* G-quadruplex is one of the most widely investigated. The *MYC* gene is frequently deregulated in cancers [13] and contains a G-quadruplex motif in the nuclease hypersensitive element (NHE) III_1_ region, located −142 to −115 bp upstream of P1 promoter which controls up to 90% of transcription [14,15]. MYC protein is a transcription factor that promotes tumorigenesis via regulation of the cell cycle, apoptosis, cell proliferation and angiogenesis (reviewed in [16]).

The NHE III_1_ region of *MYC* contains more than four guanine tracts required for intramolecular G-quadruplex formation, enabling multiple G-quadruplex topologies. The 27-nt long G-rich oligonucleotide Pu27 from the *MYC* NHE III_1_ region can form two major types of intramolecular G-quadruplexes with 1:2:1 or 1:6:1 loop arrangements, depending on which guanine tracts are involved in G-tetrad formation [17,18]. Various truncated and substituted variants of Pu27 have also been shown to form G-quadruplexes [19–21]. The Pu22 sequence lacking the 5′ terminal guanine tract, forms a parallel G-quadruplex structure in K^+^ solutions as revealed by NMR spectroscopy [18]. It has been suggested that under the conditions of negative superhelicity, the NHE III_1_ region can locally unwind and subsequently adopt a G-quadruplex/i-motif structure which can modulate protein binding and regulate gene expression [22]. Down-regulation of *MYC* gene has been observed after addition of the G-quadruplex stabilizing drug, 5,10,15,20-tetra(*N*-methyl-4-pyridyl)porphine (TMPyP4) [5,6].

Identification of G-quadruplex interacting proteins provides an opportunity for elucidating possible G-quadruplex functions *in vivo*. To this day, various proteins have been shown to interact with the *MYC* NHE III_1_ G-quadruplex. Nucleolin, a multifunctional protein localized predominantly in the nucleolus, binds specifically the *MYC* G-quadruplex, stabilizes it and this leads to suppressed *MYC* expression [9,23]. Other identified *MYC* G-quadruplex interacting human proteins include nucleoside diphosphate kinase 2 (NM23-H2) [24,25], poly(ADP-ribose) polymerase 1 (PARP-1) [26], cellular nucleic acid-binding protein (CNBP) [27], Pif1 [28] and nucleophosmin [29] though their function in *MYC* gene regulation is still not fully understood.

P53 protein is a major tumour suppressor that takes part in processes including apoptosis, DNA repair, cell cycle regulation and senescence (reviewed in [30]). The N-terminal region of p53 (aa 1–97) contains two transactivation domains (TADs) and a proline-rich region (PRR). The central region is formed by a DNA binding domain (DBD, aa 102–292) and a C-terminal region consisting of an oligomerization domain (OD, aa 323–356) and a C-terminal basic DNA binding domain (CTBD, aa 363–382, [31]). P53 can regulate the expression of its target genes directly via binding to specific p53 response elements. Sequence-specific DNA binding of p53 to its consensus sequences (p53CONs) [32] is mediated by the central DBD [33]. Wild-type p53 (wtp53) interacts with a variety of non-canonical DNA structures including three-stranded junctions [34], Holliday junctions [35], cruciforms [36], T-loops [37], stem-loops [38] and hairpins [39,40].

In the present study, we investigated wtp53 protein interaction with the *MYC* promoter G-quadruplex as this may participate in p53-mediated transcriptional regulation.

## MATERIALS AND METHODS

### DNA oligonucleotides and plasmids

Oligonucleotides were purchased from VBC-Biotech (Vienna, Austria). Sequences are presented in Table 1. Recombinant plasmids encoding human p53 proteins pT7-7wtp53 (aa 1–393), pT7-7p53CΔ30 (aa 1–363), pGEX-4TGSTp53CD (aa 94–312), pGEX-2TKGSTp53CT (aa 320–393) and pGEX-2TKGSTp53T (aa 363–393) have been described previously [41]. Supercoiled plasmids pBluescript II SK (–) (pBSK, Stratagene), pPGM1 (containing p53CON sequence AGACATGCCTAGACATGCCT) [42] and pBMYC were isolated from bacterial strain TOP10 (Stratagene) and verified by sequencing. Non-specific competitor plasmid pBSK/EcoRV was prepared by EcoRV restriction endonuclease (New England Biolabs) cleavage of pBSK. Plasmid pBMYC containing 112 bp region of the *MYC* promoter comprising NHE III_1_ sequence was constructed by cloning the 141 bp EcoRI/HinIII restriction fragment of pNHE plasmid [43] into the EcoRI/HinIII site of pBSK. Plasmid for luciferase reporter assay (pGL4-MYCII, kindly provided by Dr L. Trantírek) was constructed by cloning fragment 2292 bp upstream of *MYC* TSS into the SacI/BglII site of the pGL4.17 vector (Promega).

**Table 1:** Sequences of oligonucleotides used in this work

Label	5′ to 3′ sequence
Pu52	TTGGGGCGCTTATGGGGAGGGTGGGGAGGGTGGGGAAGGTGGGGAGGAGACT
Py52	AGTCTCCTCCCCACCTTCCCCACCCTCCCCACCCTCCCCATAAGCGCCCCAA
Pu33	TGGGGAGGGTGGGGAGGGTGGGGAAGGTGGGGA
Pu22	TGAGGGTGGGGAGGGTGGGGAA
P1-50F	GACGGTATCGATAAGAGACATGCCTAGACATGCCTCTTGATATCGAATTC
P1-50R	GAATTCGATATCAAGAGGCATGTCTAGGCATGTCTCTTATCGATACCGTC
P1-40F	GATCGATAAGAGACATGCCTAGACATGCCTCTTGATATCG
P1-40R	CGATATCAAGAGGCATGTCTAGGCATGTCTCTTATCGATC
P1-30F	GTAAGAGACATGCCTAGACATGCCTCATCG
P1-30R	CGATGAGGCATGTCTAGGCATGTCTCTTAC
P1-22F	GAGACATGCCTAGACATGCCTC
P1-22R	GAGGCATGTCTAGGCATGTCTC
A50	AAAAAAAAAAAAAAAAAAAAAAAAAAAAAAAAAAAAAAAAAAAAAAAAAA
A25	AAAAAAAAAAAAAAAAAAAAAAAAA

### p53 recombinant protein purification

Recombinant human p53 proteins: full-length wtp53 (aa 1–393), p53CΔ30 (aa 1–363), GSTp53CD (aa 94–312), p53-320 (aa 320–393), GSTp53CT (aa 320–393) and GSTp53T (aa 363–393) were expressed in *E. coli* strain C41 (DE3) and purified according to a protocol described previously [41]. The purity and size of proteins was analysed by Coomassie Brilliant Blue staining of 12.5% SDS-PAGE gels.

### EMSA in polyacrylamide gels

Oligonucleotides were radioactively 5′ end labelled with [γ-^32^P]-ATP using T4 polynucleotide kinase (New England Biolabs). G-rich oligonucleotides Pu52, Pu33 and Pu22 were heated to 95°C for 5 min in 5 mM Tris pH 7.6 and allowed to cool down gradually to room temperature in the presence of 50 mM KCl to adopt a G-quadruplex structure. Proteins (3–400 ng) were incubated with 0.25–1 pmol of radioactively labelled DNA for 20 min on ice in binding buffer (5 mM Tris pH 7.6, 0.5 mM EDTA, 0.01% Triton X-100, 50 mM KCl, 0.5 mM DTT) with 50 ng BSA and 2.5–20 ng of non-specific competitor pBSK/EcoRV. Samples were loaded on to a 6% non-denaturing polyacrylamide gel and separated by electrophoresis in 0.5× TBE buffer supplemented with 50 mM KCl. Gels were dried, exposed on a storage phosphor screen and DNA was detected using Typhoon FLA 9000 (GE Healthcare).

### CD spectroscopy

Oligonucleotides were diluted in 5 mM Tris pH 7.6 to 1–2 μM concentration. KCl was added to 10–50 mM final concentration and CD spectra were recorded after each addition at 20°C. Wtp53 protein was added to oligonucleotides in wtp53 monomer/DNA strand molar ratios 1–4 and CD spectra were recorded at 4°C. CD measurements were performed on Jasco J-815 spectropolarimeter in 10 mm Hellma microcells in the wavelength range 210–330 nm, with a scanning speed of 100 nm/min. CD spectra shown, represent the average of four scans. Molar CD values are referenced to one DNA strand.

### DMS footprinting

Oligonucleotides were radioactively 5′ end labelled with [γ-^32^P]-ATP using T4 polynucleotide kinase (New England Biolabs), heated to 95°C for 5 min and allowed to cool down gradually to room temperature either in water or in 5 mM Tris pH 7.6, 50 mM KCl. Oligonucleotides (250000 cpm, 1.7 pmol Pu52, 2.7 pmol Pu22) were subjected to dimethyl sulfate (DMS) treatment (diluted 1:400 v/v) for 5 min at room temperature. The reaction was stopped by addition of stop buffer (3 M sodium acetate:water:2-mercaptoethanol, 6:8:1, v/v). Samples were ethanol-precipitated, freeze-dried and subjected to piperidine cleavage at 90°C for 30 min. After two steps of dissolving in water and freeze-drying, the samples (80000 cpm) were loaded on to a 20% denaturing polyacrylamide gel and electrophoresed at 45 W for 1.5–3 h. Gels were exposed on a storage phosphor screen and DNA was detected using Typhoon FLA 9000 (GE Healthcare).

### EMSA in agarose gels

Supercoiled DNAs (scDNAs; 200 ng pBSK, pPGM1, pBMYC) were preincubated 50 mM KCl at 37°C for 30 min. DNAs were mixed with p53 proteins in p53 tetramer/DNA molar ratios 0.75–3 and incubated in binding buffer (5 mM Tris pH 7.6, 0.5 mM EDTA, 0.01% Triton X-100, 50 mM KCl, 0.5 mM DTT) for 30 min on ice to reach equilibrium. Samples were loaded on to a 1% agarose gel containing 0.33× TBE buffer. After 5 h electrophoresis (at 4–6 V/cm^2^), agarose gels were stained with ethidium bromide (EtBr) and photographed, more details in [44]. Intensities of bands of free scDNA substrates were quantified using ImageQuant software. Graphs show the evaluation of p53-DNA binding as the dependence of % of bound scDNA on the amount of p53 proteins (expressed as molar ratio p53/DNA). Mean values of three independent experiments were plotted on the graph.

### AFM

AFM measurements were performed on MultiMode VIII system (Veeco) with Scan-Asyst-Air tips (Bruker) in Scan Asyst in Air Mode. Plasmid pBMYC was incubated in 50 mM KCl at 37°C for 30 min and 2 ng of plasmid diluted in AFM buffer (5 mM KCl, 5 mM Hepes pH 7.6, 4 mM MgCl_2_) was deposited on a freshly cleaved mica surface, incubated for 2 min, rinsed with water and dried quickly under a stream of compressed air. For wtp53-pBMYC complex formation, protein was incubated with pBMYC plasmid in wtp53 monomer/DNA molar ratio 20/1 in binding buffer (5 mM Tris pH 7.6, 0.5 mM EDTA, 0.01% Triton X-100, 50 mM KCl) beforehand.

### Immunoprecipitation assay

Wtp53 (50 ng) was incubated with DO1 antibody (anti-p53, 400 ng) in binding buffer (5 mM Tris pH 7.6, 0.5 mM EDTA, 0.01% Triton X-100, 50 mM KCl) for 20 min on ice. ScDNA (200 ng) was then added and the samples were incubated for an additional 30 min on ice. Magnetic beads (washed three times in binding buffer beforehand) coated with protein G (DBG, Dynal/Invitrogen) were added to DO1-wtp53-DNA complexes and incubated for 30 min at 10°C. Magnetic beads were then washed with binding buffer (with 50 mM KCl) once and then twice with binding buffer containing 50–600 mM KCl. DNA was released from the beads by heating at 65°C in 1% SDS for 5 min and analysed by agarose gel electrophoresis. Intensities of bands of bound scDNA substrates were quantified using ImageQuant software. The graphs show the evaluation of p53-DNA binding as % of bound scDNA in relation to concentration of KCl. Mean values of three independent experiments were plotted on the graph.

### Human cell lines, transfections and luciferase assays

Human cell lines H1299 (p53-null, NCI-H1299, CRL-5803, A.T.C.C.), HCT116 (wtp53, p53+/+, CCL-247, A.T.C.C.), HCT116 (p53−/−, [45]) and wtp53-expressing inducible TO cell line Hwtp53 (established by protocol described in [46]) were grown in Dulbecco's modified Eagle medium (DMEM; Biosera) supplemented with 5% FBS and penicillin/streptomycin (PAA). All cultures were incubated at 37°C with 5% CO_2_. H1299 cells were seeded in 24-well plates 24 h before transfection, for luciferase assay. Cells were transfected using Lipofectamine (Invitrogen) according to the manufacturer's instructions, at 80% confluence. Construct pGL4-MYCII was used as reporter, pGL4.17 vector (Promega) was used as control and the linear form was prepared by BamHI restriction enzyme cleavage. Plasmid pRL-SV40 (20 ng) encoding the *Renilla* luciferase, was used as a control for transfection efficiency. Where appropriate, 150 ng of the p53 expression vector based on pCDNA3.1p53 [44] or empty vector pCDNA3.1 was co-transfected with 400 ng of reporter constructs pGL4-MYCII or pGL4.17 in supercoiled or linear form of reporter. Approximately 48 h after transfection, extracts were prepared using the Dual Luciferase Assay System (Promega) following the manufacturer's protocol and luciferase activities were measured in a plate reader luminometer Immunotech LM-01T. For each construct, relative luciferase activity is defined as the mean value of the firefly luciferase/*Renilla* luciferase ratios for pCDNA3.1p53 effector divided by the mean value for pCDNA3.1 vector only, results were obtained from three independent experiments.

### Western blot analysis

H1299, HCT116 (p53+/+), HCT116 (p53−/−) and Hwtp53 (expressing wtp53, induced with 1 μg/ml tetracycline for 20 h) [44] cells were harvested from 10 cm plates and lysed with 1× PLB (Promega), followed by the sonication of cells (Bandelin Sonopuls). Samples (100 μg of total protein) were analysed on 12.5% SDS-PAGE gels and proteins were detected by the following primary antibodies: DO1 (anti-p53, kindly provided by B. Vojtesek), anti-cyclin-dependent kinase inhibitor 1A (CDKN1A; Millipore), anti-β-Actin (Sigma), anti-BAX (Sigma), anti-MYC (Cell Signaling).

### ChIP assay

HCT116 (p53+/+), HCT116 (p53−/−), H1299 (p53 null) and Hwtp53 cells were cross-linked with formaldehyde and subjected to ChIP assays as previously described [44] with the following modifications: the sonication of cells was limited to 4 kJ (Bandelin Sonopuls). Purified antibodies DO1 and IgG were incubated overnight with diluted chromatin and immunoprecipitations were performed with protein G magnetic beads (Invitrogen). The PCR was performed using primers targeting *MYC* NHE III_1_ (myc-chipTQF: CTACGGAGGAGCAGCAGAGAA; myc-chipTQR: GCCTCTCGCTGGAATTACT). For quantitative analysis, PCR was carried out for 25 cycles.

## RESULTS

### Wild-type p53 binds *MYC* parallel G-quadruplex

**Figure 1:**
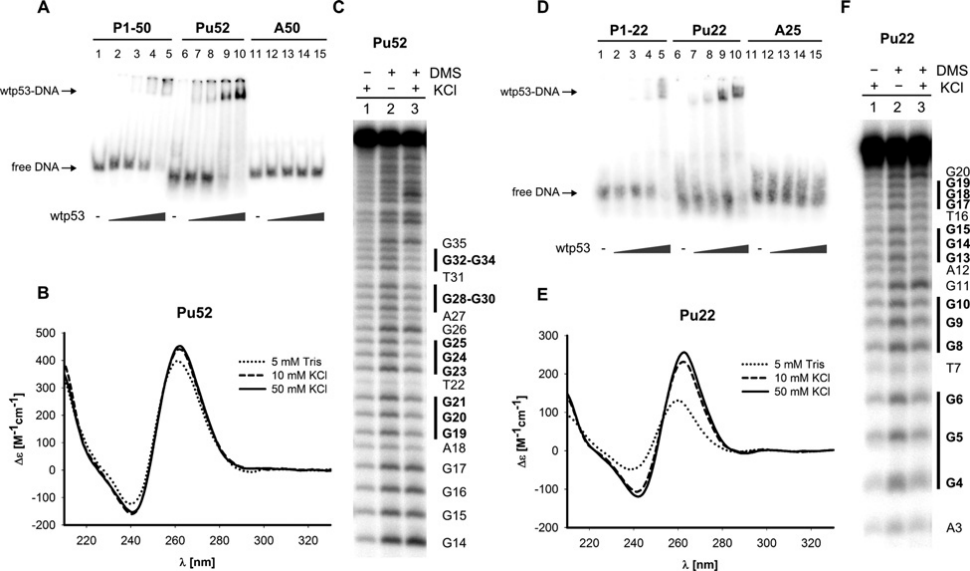
Full-length wild-type p53 binding to the parallel G-quadruplex from *MYC* promoter NHE III_1_ region is comparable with p53CON binding (**A**) Comparison of sequence-specific and *MYC* G-quadruplex Pu52 binding of p53 by EMSA. Oligonucelotides P1-50 (0.25 pmol, lanes 1–5), Pu52 (1 pmol, lanes 6–10) and A50 (0.25 pmol, lanes 11–15) were incubated with wtp53 protein (50, 100, 200, 400 ng/1 pmol of oligonucleotide) in the presence of 20 ng (per 1 pmol of oligonucleotide) of non-specific competitor pBSK/EcoRV. (**B**) CD spectra of Pu52 oligonucleotide measured in 5 mM Tris pH 7.6 (dotted line) and after addition of 10 mM KCl (dashed line) and 50 mM KCl respectively (solid line). (**C**) DMS footprinting of Pu52 oligonucleotide. Pu52 was annealed in 50 mM KCl without subsequent DMS treatment (lane 1), annealed in the absence of KCl and treated with DMS (lane 2) or annealed in 50 mM KCl and treated with DMS (lane 3). (**D**) Comparison of sequence-specific and *MYC* G-quadruplex Pu22 binding of p53 by EMSA. Oligonucelotides P1-22 (0.25 pmol, lanes 1–5), Pu22 (1 pmol, lanes 6–10) and A25 (0.25 pmol, lanes 11–15) were incubated with wtp53 protein (50, 100, 200, 400 ng/1 pmol of oligonucleotide) in the presence of 20 ng (per 1 pmol of oligonucleotide) of non-specific competitor pBSK/EcoRV. (**E**) CD spectra of Pu22 oligonucleotide measured in 5 mM Tris pH 7.6 (dotted line) and after addition of 10 mM KCl (dashed line) and 50 mM KCl respectively (solid line). (**F**) DMS footprinting of Pu22 oligonucleotide. Pu22 was annealed in 50 mM KCl without subsequent DMS treatment (lane 1), annealed in the absence of KCl and treated with DMS (lane 2) or annealed in 50 mM KCl and treated with DMS (lane 3).

To investigate wtp53 binding to G-quadruplex DNA by EMSA, G-quadruplexes formed by single-stranded oligonucleotides Pu52, Pu33 and Pu22 derived from the NHE III_1_ region of the *MYC* gene promoter were selected. G-quadruplexes were formed by heating the G-rich oligonucleotides to 95°C and annealing them in 50 mM KCl. Full-length wtp53 binds the G-quadruplex Pu52 with an affinity comparable to double-stranded P1-50 oligonucleotide which includes 20 bp p53CON (Figure 1A). Wtp53 also binds the G-rich sequences Pu22 (Figure 1D) and Pu33 (Supplementary Figure S1A) with an affinity comparable to the shorter double-stranded p53CON containing oligonucleotides P1-22 and P1-30 respectively. Wtp53 affinity to Pu52 is higher than to Pu22 and Pu33. Oligonucleotides A50 (Figure 1A) and A25 (Figure 1D) are not bound by wtp53 under the same conditions. G-quadruplex formation of the *MYC* NHE III_1_ region derived oligonucleotides was examined by CD spectroscopy and DMS footprinting. CD spectra of Pu52, Pu33 and Pu22 exhibit a positive peak at 260 nm and negative peak at 240 nm characteristic of a parallel G-quadruplex structure (Figures 1B, 1E and Supplementary Figure S1B) that is stabilized after addition of 10 and 50 mM KCl. DMS footprinting of Pu52 (Figure 1C) and Pu22 (Figure 1F) revealed a partial blocking of DNA cleavage in four consecutive G-tracts after annealing of oligonucleotides in 50 mM KCl, suggesting a G-quadruplex formation with 1:2:1 loop arrangement. Furthermore, we studied the effect of wtp53 on *MYC* G-quadruplex structure by CD spectroscopy. Addition of wtp53 to *MYC* G-quadruplexes Pu52, Pu33 and Pu22 folded in 50 mM KCl resulted in preserved positive peak at 260 nm, suggesting that wtp53 does not unwind the *MYC* parallel G-quadruplex (Supplementary Figures S1C and S2).

### Full-length wild-type p53 binds *MYC* G-quadruplex with higher affinity than its isolated C-terminal region or central DNA binding domain

**Figure 2:**
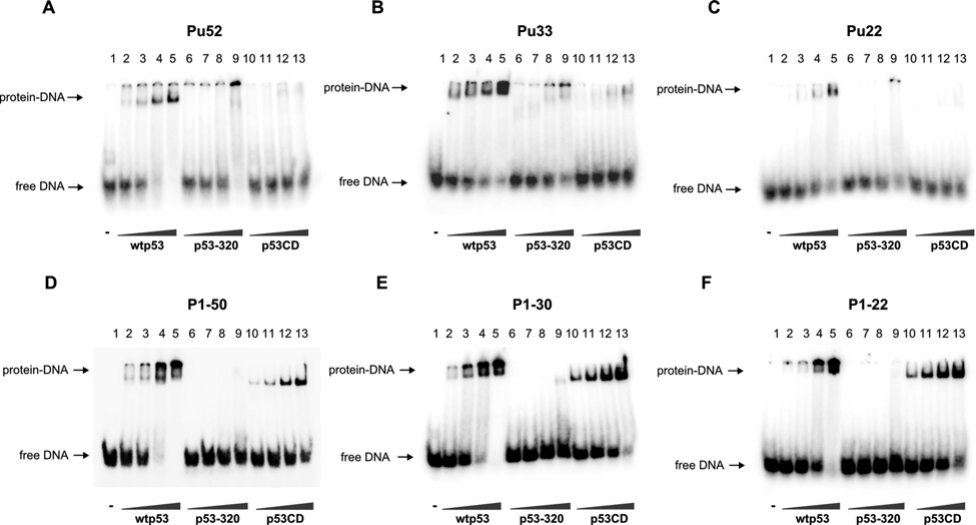
Full-length wild-type p53 binds to *MYC* G-quadruplex more efficiently than its isolated C-terminal and central regions The role of p53 domains in *MYC* G-quadruplex binding was studied by EMSA. Oligonucleotides (1 pmol) Pu52 (**A**), Pu33 (**B**) and Pu22 (**C**) were incubated with wtp53 (lanes 2–5; 50, 100, 200, 400 ng), p53-320 (lanes 6–9; 25, 50, 100, 200 ng) or p53CD (lanes 10–13; 12.5, 25, 50, 100 ng) in the presence of 10 ng of non-specific competitor pBSK/EcoRV. Binding of full-length wtp53, central DBD and C-terminal construct of p53 to double-stranded oligonucleotides containing p53CON was studied by EMSA. Oligonucleotides (0.25 pmol) (**D**) P1-50, (**E**) P1-30 and (**F**) P1-22 were incubated with wtp53 (lanes 2–5; 12.5, 25, 50, 100 ng), p53-320 (lanes 6–9; 6, 12.5, 25, 50 ng) or p53CD (lanes 10–13; 3, 6, 12.5, 25 ng) in the presence of 2.5 ng of non-specific competitor pBSK/EcoRV.

The ability of full-length wtp53 protein to bind *MYC* G-quadruplex was compared with isolated central DBD (p53CD, aa 94–312) and C-terminal construct containing OD and basic domain (p53-320, aa 320–393). Wtp53 binds the Pu52 (Figure 2A), Pu33 (Figure 2B) and Pu22 (Figure 2C) G-quadruplexes with higher affinity than p53-320 and p53CD proteins. Double-stranded oligonucleotides P1-50, P1-30 and P1-22 containing the 20 bp p53CON sequence are preferentially bound by full-length wtp53 and central DBD of p53, whereas p53-320 protein exhibits only minimal binding (Figures 2D–2F). In both sets of DNA substrates, protein binding is strengthened with increasing oligonucleotide length.

### Full-length wild-type p53 and the C-terminal region of p53 specifically recognize the G-quadruplex over double-stranded *MYC* NHE III_1_

**Figure 3:**
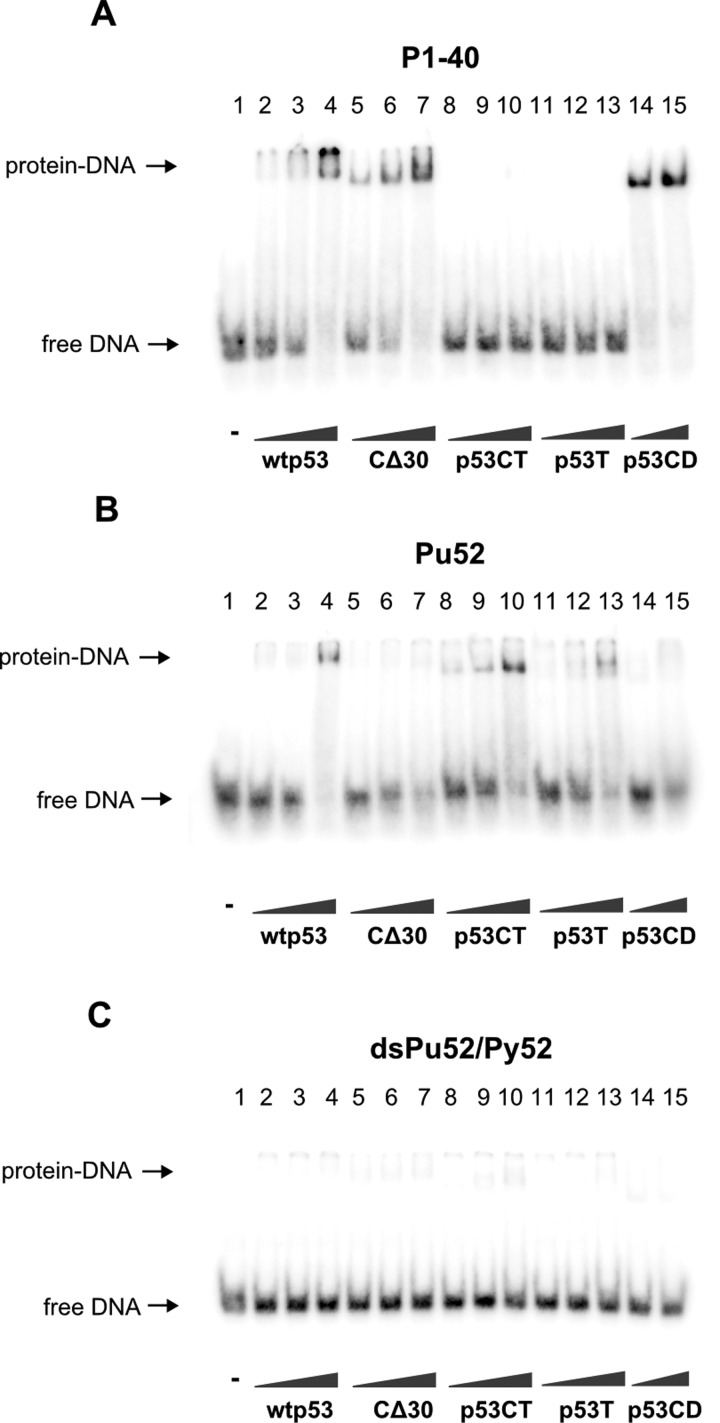
Wild-type p53 and C-terminal region of p53 bind G-quadruplex Pu52 with higher affinity than double-stranded Pu52/Py52 Binding of various p53 protein constructs to *MYC* promoter G-quadruplexes from was studied by EMSA. Oligonucleotides (**A**) P1-40 (0.5 pmol), (**B**) Pu52 (1 pmol) and (**C**) dsPu52/Py52 (0.5 pmol) were incubated with wtp53 (lanes 2–4; 25, 50, 100 ng/1 pmol of oligonucleotide), CΔ30 (lanes 5–7; 25, 50, 100 ng/1 pmol of oligonucleotide), p53CT (lanes 8–10; 50, 100, 200 ng/1 pmol of oligonucleotide), p53T (lanes 11–13; 50, 100, 200 ng/1 pmol of oligonucleotide) or p53CD (lanes 14 and 15; 100, 200 ng/1 pmol of oligonucleotide) in the presence of 20 ng (per 1 pmol of oligonucleotide) of non-specific competitor pBSK/EcoRV.

The role of p53 protein domains in G-quadruplex binding was further tested by EMSA with a set of p53 protein constructs. Full-length wtp53, p53 lacking the last 30 C-terminal amino acids (CΔ30) and central DBD (p53CD) bind P1-40 oligonucleotide containing p53CON (Figure 3A). G-quadruplex Pu52 was bound preferentially by wtp53 and the C-terminal region of p53 (p53CT, aa 320–393), to a lesser extent by protein construct comprising only the last 30 C-terminal amino acids (p53T, aa 363–393). p53CD and CΔ30 proteins exhibit only minimal binding to Pu52 (Figure 3B). All tested proteins exhibited only residual binding to double-stranded dsPu52/Py52, formed by complementary G-rich Pu52 and C-rich Py52 oligonucleotides, containing NHE III_1_ sequence (Figure 3C).

### Wild-type p53 binds supercoiled pBMYC plasmid containing NHE III_1_ sequence

**Figure 4:**
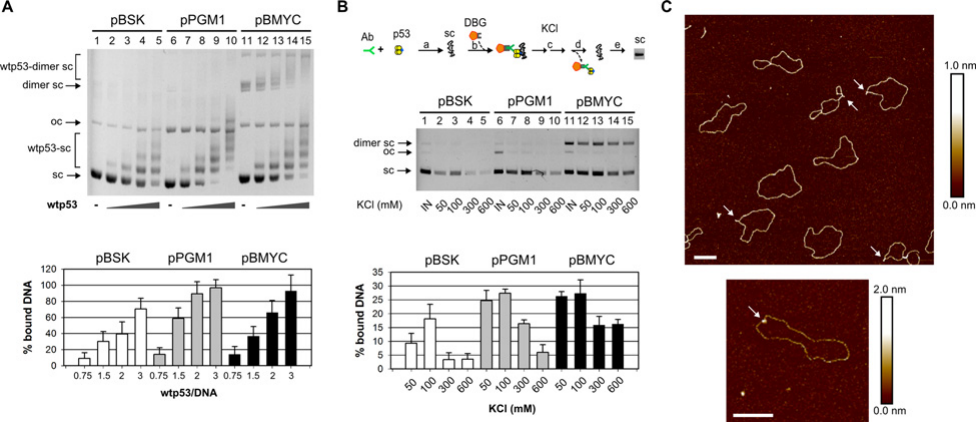
Wild-type p53 tightly binds supercoiled plasmid pBMYC containing the *MYC* NHE III_1_ sequence (**A**) Wtp53 binding to supercoiled plasmids pBSK, pPGM1 and pBMYC was studied by EMSA in agarose gel. Wtp53 protein was incubated with scDNA (pBSK, 200 ng, lanes 1–5), scDNA with CON (pPGM1, 200 ng, lanes 6–10) and scDNA with *MYC* G-quadruplex forming sequence (pBMYC, 200 ng, lanes 11–15) in p53/DNA molar ratios 0.75, 1.5, 2 and 3 at 4°C, EMSA was performed at 4°C. Graphs show the evaluation of p53-DNA binding as the dependence of percents of bound scDNA (*y*-axis) on the amount of p53 proteins (expressed by molar ratio p53/DNA, *x*-axis). Mean values of three independent experiments were plotted on the graph, representative gel is shown on the top. (**B**) Wtp53 binding to supercoiled plasmids pBSK, pPGM1 and pBMYC studied by immunoprecipitation on magnetic beads (scheme describes performed procedure). After binding of wtp53–DNA complexes on magnetic beads, they were washed in buffer containing 50–600 mM KCl. Arrows indicate precipitated supercoiled (sc), open circular (oc) and supercoiled dimers (dimer sc). Mean values of bound scDNA from three independent experiments were plotted in the graph, representative gel is shown in the middle section. (**C**) Visualization of pBMYC plasmid (top, line bar represents 200 nm) and wtp53–pBMYC complex (bottom, line bar represents 200 nm) by AFM on air. Potential G-quadruplex formation was considered from the shape of the molecules, positions of potential G-quadruplex structures are indicated by arrows on the top section. The complex of wtp53-pBMYC is indicated by arrow on the bottom section.

First we compared binding of wtp53 to scDNAs at native superhelical density. These scDNAs contain either G-quadruplex forming sequence (pBMYC) or specific p53CON sequence (pPGM1). Differences in wtp53 recognition of scDNA with and without G-quadruplex forming sequence are measurable by number and intensity of retarded bands (compare lanes 5 and 15, Figure 4A). Both plasmids pPGM1 (with CON, lanes 7–10) and pBMYC (lanes 12–15) were more strongly bound by p53 than pBSK (lanes 2–5, Figure 4A). The stability of wtp53 binding to scDNA pPGM1 (p53CON) and pBMYC (*MYC* promoter sequence) was compared by immunoprecipitation assay. Wtp53 was incubated with supercoiled plasmids and bound via DO1 antibody on magnetic beads in binding buffer containing 50 mM KCl. To investigate the stability of formed protein–DNA complexes, these were subjected to two-step washing in buffer containing various concentrations of KCl. Binding of wtp53 to supercoiled pBSK control plasmid observed after washing with 50 mM and 100 mM KCl, was dramatically abolished when the washing buffer contained 300 mM or 600 mM KCl (Figure 4B). Decreased wtp53 binding after washing in 300 mM and 600 mM KCl was also observed for pPGM1 and to a lesser extent for pBMYC plasmid (Figure 4B). These data suggest that wtp53 binding to scDNA is more stable if the plasmid includes the *MYC* promoter region containing NHE III_1_ sequence or p53CON. pBMYC plasmid was visualized by AFM either alone (Figure 4C, top) or in complex with wtp53 protein (Figure 4C, bottom). Potential G-quadruplex formation was considered from the shape of the molecules.

### Wild-type p53 represses and binds *MYC* promoter *in vivo*

Several reports have demonstrated that wtp53 represses the transcription of *MYC in vivo* [47–50]. To confirm the involvement of wtp53 binding to *MYC* promoter G-quadruplex in this regulation, we used luciferase reporter assay and ChIP techniques.

**Figure 5:**
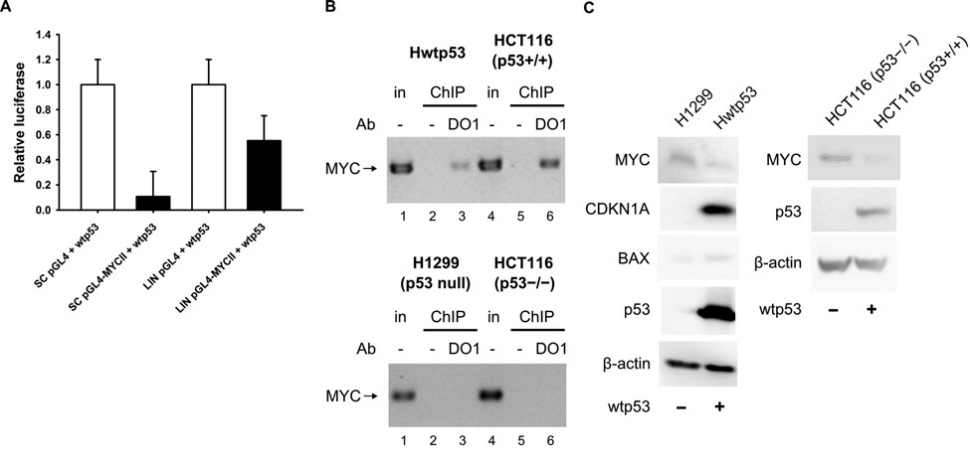
Wtp53 represses *MYC* promoter activity and binds to *MYC* promoter *in vivo* (**A**) Influence of DNA topology on wtp53-driven repression of *MYC* promoter. Luciferase assay showing stronger repression of *MYC* promoter in supercoiled pGL4-MYCII plasmid by wtp53 in contrast to linear plasmid pGL4-MYCII/BamHI. Supercoiled pGL4.17 and linear pGL4.17/BamHI were used as control vectors. Mean values of relative luciferase assay (normalized on *Renilla* luciferase) from three independent experiments were plotted on the graph. (**B**) ChIP showing wtp53 binding to *MYC* promoter which contains a G-quadruplex motif. DNA fragments were immunoprecipitated using DO1 antibody against p53 in Hwtp53 (top, lane 3) and HCT116 (p53+/+) (top, lane 6) cells, negative control ChIP without Ab (lanes 2 and 5), positive input control (1/15 input for ChIP, lanes 1 and 4). The same procedure was performed in p53 null cell lines H1299 and HCT116 (p53−/−). (**C**) Wtp53 mediated down-regulation of MYC at the protein level and activation of BAX and CDKN1A was analysed in Hwtp53 cells compared with H1299 without p53 expression. Western blots presenting the protein levels of p53, MYC, CDKN1A, β-Actin and BAX. Wtp53 mediated down-regulation of MYC at the protein level was analysed in HCT116 (p53+/+) compared with HCT116 (p53−/−).

To analyse whether G-quadruplex-forming sequence had any effect on p53-driven transcription, we performed luciferase reporter assays using reporter vectors in variants with (pGL4-MYCII) and without (pGL4) *MYC* G-quadruplex motif (Figure 5A). Luciferase assay was performed in H1299 cells with linear and supercoiled reporter vectors and with pCDNA3.1p53 effector or pCDNA3.1 vector only (Figure 5A). Only supercoiled reporter sc pGL4-MYCII could form G-quadruplex structure. P53 expression resulted in strong repression of pGL4-MYCII vectors. Repression was stronger for sc form of reporter (Figure 5A) than lin form of reporter. These results show that DNA superhelicity possibly accompanied by G-quadruplex formation enhances *MYC* promoter repression mediated by wtp53.

To confirm wtp53 binding to *MYC* promoter *in vivo*, ChIP was performed in two cell line based models with endogenous HCT116 (p53+/+) and exogenous Hwtp53 expression and their p53 null forms HCT116 (p53−/−) and H1299 (Figure 5B). ChIP with p53 specific antibody (DO1) confirmed endogenous (Figure 5B, lane 6) and exogenous (Figure 5B, lane 3) wtp53 binding in contrast to their negative controls. Lastly, the effect of endogenous and exogenous wtp53 expression on *MYC* regulation was investigated at the protein level (Figure 5C). Suppressed level of the MYC protein in Hwtp53 (tetracycline-inducible wtp53) cells compared with H1299 (p53-null) cells correlated with wtp53 induction and activation of CDKN1A (p21) and BAX (Figure 5C). In the case of endogenous cell line HCT116 (p53+/+), the difference in MYC protein expression in comparison with HCT116 (p53−/−) was not so robust.

## DISCUSSION

Tumour suppressor p53 protein functions as a transcriptional regulator of a vast number of genes through binding to p53 response elements in the genome that may differ from the established consensus sequence motif (reviewed in [51]). The central DBD of p53 is crucial for high affinity recognition of consensus sequences [33] and the C-terminal domain was shown to regulate sequence-specific DNA binding [52]. The importance of the C-terminal region of p53 was demonstrated on a mouse model, where it affected p53-dependent gene expression in a tissue-specific manner by impairing p53 DNA binding or regulating p53 protein levels and activity [53]. Recently, it has been shown that the intact C-terminal region of p53 is important for stable p53 tetramer–DNA complex formation [54].

In the present study, we investigated the selective binding of wtp53 to the purine-rich G-quadruplex forming sequence of the *MYC* NHE III_1_ region located upstream of the P1 promoter which is believed to have a significant role in *MYC* transcriptional regulation. The topology of the NHE III_1_ can be altered by negative supercoiling and binding of various proteins or ligands [22]. Our results show that full-length wtp53 binds to the parallel G-quadruplex formed by purine-rich strand of *MYC* NHE III_1_, whereas it does not bind to the NHE III_1_ in double-stranded form. Our data suggest that wtp53 can bind *MYC* parallel G-quadruplex selectively with affinity comparable to p53CON. Using different p53 protein constructs, we found that the C-terminal region of p53 can autonomously bind to G-quadruplex with higher affinity than isolated central DBD. In our previous study, we showed that several full-length hot spot mutant p53 proteins (R273H, R248W and G245S) and wtp53 bound selectively to G-quadruplexes formed by 52-mer from *MYC* NHE III_1_ and 61-mer from telomerase reverse transcriptase (*TERT*) core promoter and that mutant p53 R273H stabilizes both *MYC* and *TERT* G-quadruplex structures [55]. Hence, the results with full-length wtp53 [55] are in good agreement with the results presented in the present study. Full-length hot spot mutant p53 proteins contain a mutated core domain but intact C-terminus, common recognition of *MYC* G-quadruplex by hot spot mutp53 and wtp53, support the conclusion from the data that the C-terminus of p53 is mainly responsible for p53 G-quadruplex recognition.

Both wtp53 and mutp53 have been described as regulating the transcription of *MYC* but in the opposite direction [47,48,50,56]. Transcriptional activation of the *MYC* gene in relation to the p53 C-terminus has been described as one of the acquired functions of mutant p53 D281G [56]. Mutant p53 binding sites on DNA in that study were not examined. Using custom tiling array that covers a total of 902 genes including putative mutant p53 target genes, we showed earlier in ChIP-chip experiments that oncogenic mutant p53 R273H preferentially and autonomously binds to CpG islands around transcription start sites of many active genes with the potential to form G-quadruplexes. However, mutant p53 binding to *MYC* promoter was not examined in cells [55].

Repression of *MYC* by wtp53 has been observed in several human and mouse cell lines and mouse tissues [47,49,57]. Association of wtp53 with *MYC* promoter may represent one of the initial steps in p53-mediated gene regulation. For this reason, we detected wtp53 binding to G-quadruplex forming sites of the *MYC* promoter in the context of chromatin by ChIP assay. Endogenous wtp53 expressed in HCT116 (p53+/+) cells and exogenous wtp53 expressed in Hwtp53 were bound to G-quadruplex, forming the *MYC* NHE III_1_ region but so far this binding has not been detected in cells. Binding of thermosensitive mutant p53 with wild-type conformation to *MYC* promoter *in vivo* was identified in DP16.1/p53ts murine cells [47]. However, the identified murine sequence, which is homologous with human NHE III_1_ region, does not share the same putative G-quadruplex motif.

The ability of p53 to suppress the expression of cell cycle regulatory and growth promoting genes, including *MYC* [57,58] via multiple mechanisms is one of the most important factors in protection from tumorigenesis. We propose that p53 binding to G-quadruplex in *MYC* promoter may be one of the steps in *MYC* repression. Transcriptional repression of *MYC* by p53 was also associated with histone deacetylation upon p53 binding [47]. Another scenario is that indirect p53-dependent *MYC* repression involves the activation of *miR-145* via binding of p53 to its potential p53 response element [59]. *MYC* promoter contains several p53 consensus motifs, p53 binding to two of these was shown by ChIP in murine cells [47]. In contrast to results in differentiated cells, in murine embryonic stem cells, p53-mediated induction of *MYC* transcription was observed [60]. The mechanisms of p53-dependent *MYC* regulation described so far lead us to hypothesize that p53 binding can potentiate the recruitment of co-regulators which may ultimately regulate transcription in the desired direction.

In conclusion, the results suggest that wtp53 protein can bind *MYC* promoter G-quadruplex and the C-terminal region of p53 is critical for G-quadruplex recognition. P53 binding to G-quadruplexes in promoter regions of p53 target genes may play a role in p53-mediated transcriptional regulation.

## Abbreviations

CDKN1A: cyclin-dependent kinase inhibitor 1A
CNBP: cellular nucleic acid-binding protein
DBD: DNA binding domain
DMS: dimethyl sulfate
NHE: nuclease hypersensitive element
OD: oligomerization domain
p53CON: p53 consensus sequence
scDNA: supercoiled DNA
*TERT*: telomerase reverse transcriptase
wtp53: wild-type p53
